# Community Nursing Interventions and Their Impact on Health Outcomes: A Systematic Review of International Evidence

**DOI:** 10.7759/cureus.89575

**Published:** 2025-08-07

**Authors:** Akiko Yata, Ryuichi Ohta, Yoshinori Ryu, Yoshiaki Iwashita, Chiaki Sano

**Affiliations:** 1 Nursing, Community Nurse Company, Unnan, JPN; 2 Community Care, Unnan City Hospital, Unnan, JPN; 3 Emergency and Critical Care Medicine, Shimane University, Izumo, JPN; 4 Community Medicine Management, Shimane University, Izumo, JPN

**Keywords:** chronic disease management, community health nursing, family medicine, general medicine, health promotion, health services accessibility, home care services, patient-centered care

## Abstract

This systematic review synthesized findings from 17 studies conducted between 2000 and 2024, focusing on the definitions, interventions, and outcomes associated with community nursing. The studies originated from diverse countries, including Singapore, Australia, Italy, Portugal, and the United States, and employed various designs such as quasi-experimental trials, pre-post evaluations, and descriptive studies. Sample sizes ranged from 23 to over 1,600 participants, with most targeting older adults or individuals with chronic conditions. Definitions of community nursing varied, reflecting differences in national healthcare systems. Still, common features included nurse-led services focused on prevention, self-management support, and care coordination in non-hospital settings. Interventions ranged from short-term health fairs to long-term, structured programs, often including health assessments, lifestyle coaching, medication adherence support, and caregiver education. Outcomes were grouped into four categories: healthcare service utilization, patient-reported outcomes, clinical indicators, and quality of life. Notably, one large-scale program in Singapore reported a 23% reduction in emergency department visits. Other studies documented increases in self-monitoring behavior, improved self-efficacy, and greater patient satisfaction. However, limitations included small sample sizes, non-randomized designs, and reliance on self-reported data. Despite these constraints, the review highlights the potential of community nursing to improve individual health outcomes and reduce healthcare system burdens.

## Introduction and background

With the growing aging population and the increasing prevalence of chronic diseases, healthcare systems worldwide are shifting from hospital-centered models to community-based integrated care [1,2]. Community nursing has emerged as a key strategy to address the complex health and social care needs of people living in the community, including older adults, individuals with chronic conditions, and those at risk of social isolation [3,4].

Community nursing refers to a broad spectrum of nurse-led services provided outside of hospital settings, encompassing health promotion, disease prevention, chronic disease management, medication support, rehabilitation, end-of-life care, family support, and coordination with local resources [5,6]. Unlike traditional clinical care, community nurses work from a lifestyle-oriented and person-centered perspective, building long-term relationships with clients to deliver continuous and holistic support within their living environments [6].

However, the term “community nursing” varies considerably across countries and healthcare systems. For instance, it may correspond to “district nursing” in the United Kingdom, “public health nursing” in Japan, or “home care nursing” in North America [5]. This conceptual heterogeneity, along with the diversity of intervention models and target populations, makes it difficult to generalize findings or compare outcomes across studies [5].

Although numerous studies have reported the benefits of community nursing interventions, these findings remain fragmented and lack integration. To date, no comprehensive systematic review has rigorously examined the global definitions, operational frameworks, and clinical effectiveness of community nursing.

Therefore, this systematic review aims to (1) clarify how community nursing is defined in different contexts, (2) describe the structure and components of interventions implemented under the banner of community nursing, and (3) evaluate their reported impacts on health outcomes, quality of life, patient satisfaction, and healthcare utilization. By synthesizing evidence from a wide range of international studies, this review aims to inform the development of practice models, policy decisions, and future research directions in community-based nursing care.

## Review

Methods

Study Design

This study is a systematic review conducted in accordance with the Preferred Reporting Items for Systematic Reviews and Meta-Analyses (PRISMA) 2020 guidelines [7]. The review was designed to synthesize existing literature on the definitions, interventions, and outcomes associated with community nursing across various international contexts.

Protocol Registration

The International Prospective Register of Systematic Reviews (PROSPERO) has registered the review protocol. The registered number was CRD420251103711.

Search Strategy

A comprehensive literature search was conducted across the following electronic databases: PubMed, CINAHL (Cumulative Index to Nursing and Allied Health Literature), Embase, and Web of Science. The search covered studies published from January 1, 2000, to May 31, 2025. Search terms included combinations of keywords and MeSH terms such as “community nursing,” “district nursing,” “public health nursing,” “home care nursing,” “intervention,” “program,” “outcomes,” “effectiveness,” and “quality of life.” Boolean operators were used to refine the search strategy. Only peer-reviewed articles written in English were included. Grey literature, conference abstracts, and non-peer-reviewed sources were excluded from the analysis. The full search strategy, including keywords, Boolean operators, years covered, and filters applied, is shown in Table 1.

**Table 1:** Search strategy for each database

Database	Search Terms	Boolean Operators	Years Covered	Filters Applied
PubMed	("community nursing"[MeSH Terms] OR "district nursing" OR "public health nursing" OR "home care nursing") AND ("intervention" OR "program" OR "model") AND ("outcome" OR "effectiveness" OR "quality of life")	AND/OR	Jan 2000-May 2025	English only, peer-reviewed
CINAHL	("community nursing" OR "district nursing" OR "home care nursing" OR "public health nursing") AND ("health promotion" OR "intervention" OR "program") AND ("outcome" OR "patient satisfaction" OR "hospitalization")	AND/OR	Jan 2000-May 2025	English only, research articles
Embase	('community nursing'/exp OR 'district nursing'/exp OR 'public health nursing'/exp OR 'home care nursing'/exp) AND ('intervention' OR 'health program' OR 'care model') AND ('outcome' OR 'healthcare utilization' OR 'quality of life')	AND/OR	Jan 2000-May 2025	English only, human studies
Web of Science	TS=("community nursing" OR "district nursing" OR "home care nursing" OR "public health nursing") AND TS=(intervention OR program OR model) AND TS=(outcome OR effectiveness OR "quality of life")	AND	Jan 2000-May 2025	English only, peer-reviewed

Study Selection

Eligible studies included original research articles that (1) focused on the definition or implementation of community nursing, (2) evaluated specific nursing interventions delivered in community settings, and (3) reported on at least one health-related, psychosocial, or healthcare utilization outcome. Studies involving hospital-based nursing practice, editorials, commentaries, case reports, and protocols without primary data were excluded. Two independent reviewers (RO and YR) screened the titles and abstracts of retrieved articles, followed by a full-text review of potentially eligible studies. Discrepancies were resolved through discussion with a third reviewer (AY).

Data Extraction and Synthesis

Data extraction was performed using a standardized form. The following information was collected: author(s), publication year, country, study design, sample size, participant characteristics, definitions of community nursing and community nurse, description of the intervention (including duration, frequency, provider, and components), outcome measures, main results, and study limitations. Extracted data were tabulated and narratively synthesized to highlight patterns in definitions, intervention strategies, and observed effects. Thematic synthesis was conducted for qualitative findings. When applicable, intervention components and outcome categories were grouped for comparative analysis.

Statistical Analysis

Where studies reported quantitatively comparable outcomes, such as changes in quality-of-life scores, hospitalization rates, or patient satisfaction metrics, meta-analyses were planned using either fixed-effects or random-effects models, depending on the level of heterogeneity. Continuous outcomes were summarized using mean differences (MD) or standardized mean differences (SMD) with 95% CIs. Dichotomous outcomes were reported as risk ratios (RRs) or odds ratios (ORs) with corresponding 95% CIs. Heterogeneity was assessed using the I² statistic and chi-squared test, with an I² value greater than 50% indicating substantial heterogeneity. Publication bias was examined using funnel plots and Egger’s test if more than 10 studies were included in the analysis.

Risk of Bias Assessment

The methodological quality of the included non-randomized studies was assessed using the ROBINS-I (Risk Of Bias In Non-randomized Studies - of Interventions) tool, developed by the Cochrane Bias Methods Group. This tool evaluates seven domains of bias: confounding, participant selection, intervention classification, deviations from intended interventions, missing data, outcome measurement, and the selection of reported results. Each domain was rated as low, moderate, serious, or critical risk of bias following the guidance in the ROBINS-I manual. Two reviewers (RO and YR) independently performed the assessments, and discrepancies were resolved by discussion with a third reviewer (AY). The ROBINS-I tool is publicly available under open-access licensing from the Cochrane Bias Methods Group and does not require explicit permission for academic use.

Results

Study Selection

**Figure 1:**
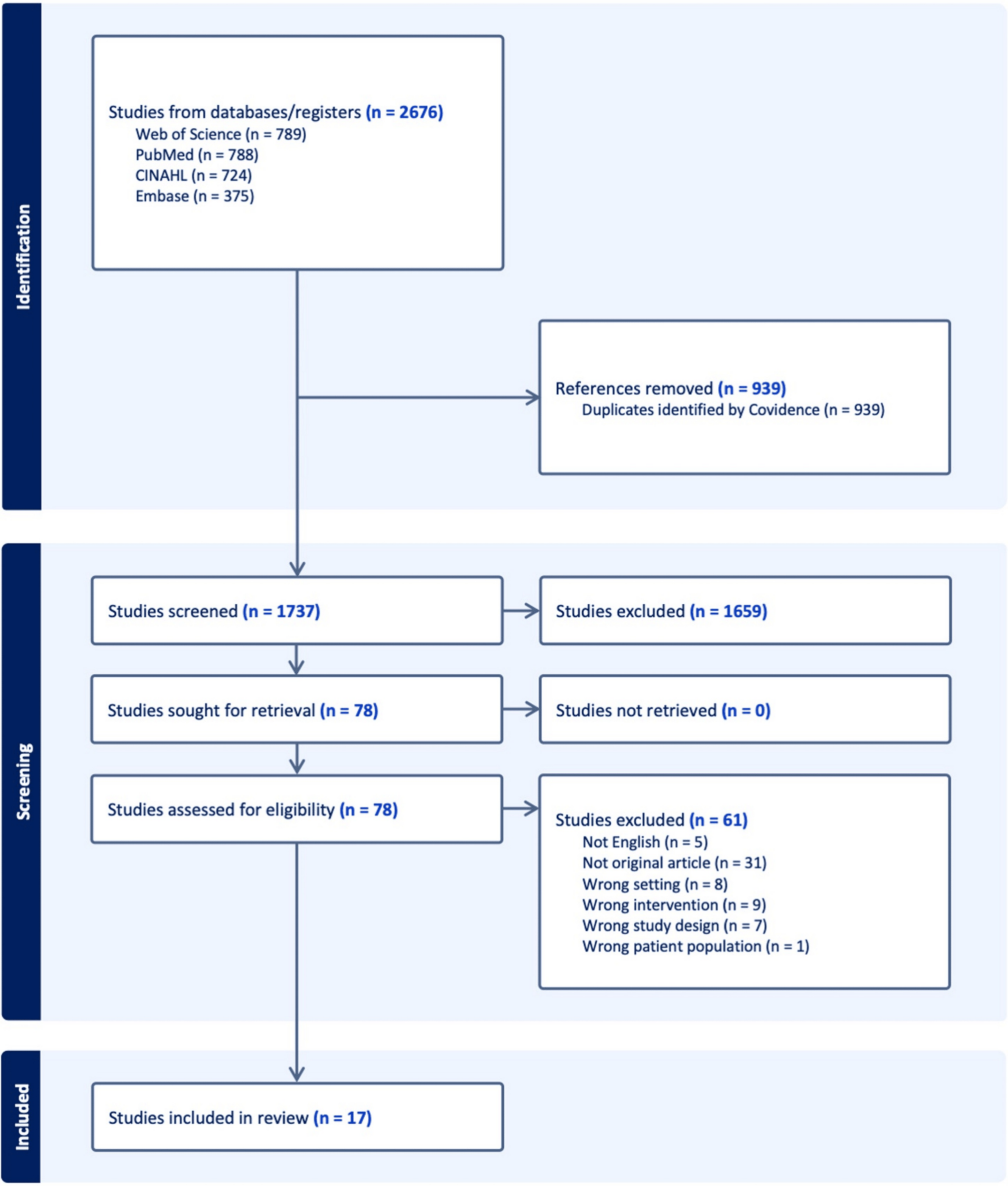
Selection flow

A total of 2,676 records were identified through electronic database searches: Web of Science (n = 789), PubMed (n = 788), CINAHL (n = 724), and Embase (n = 375). After removing 939 duplicates using Covidence, 1,737 records remained for title and abstract screening. Of these, 1,659 studies were excluded during the screening phase. A total of 78 full-text articles were assessed for eligibility. After full-text review, 61 articles were excluded for the following reasons: not an original article (n = 31), wrong intervention (n = 9), wrong setting (n = 8), wrong study design (n = 7), not in English (n = 5), and wrong patient population (n = 1). Ultimately, 17 studies met the eligibility criteria and were included in this systematic review. No studies were excluded due to retrieval issues. The full study selection process is illustrated in the PRISMA 2020 flow diagram (Figure 1).

Characteristics of the Included Articles

The included studies were published between 2000 and 2024 and originated from various countries, including Singapore, Australia, Portugal, and the United States. Study designs varied and included quasi-experimental trials, single-group pretest-posttest studies, descriptive evaluations, and cross-sectional studies. Sample sizes ranged from as few as 23 participants to over 1,600 individuals. Most studies focused on older adults, individuals with chronic conditions, or community-dwelling populations at risk of social isolation or poor health outcomes. A summary of study characteristics is provided in Table 2.

**Table 2:** Characteristics of included studies Participant age and sex distributions are reported as described in each original study. Where not explicitly stated, estimates were made based on contextual information provided by the authors. RCT, randomized controlled trial; Q-E, quasi-experimental; pre-post, pretest-posttest design; NR, not reported

Author (Year)	Country	Study Design	Sample Size	Age of Participants	Sex Distribution
M. Flanagan (2001) [8]	United Kingdom	Qualitative study	15 district nurses (focus groups), 15 social service home carers (focus groups), 12 patients with healed venous leg ulcers (semi-structured interviews)	District nurses: 25-57 years (mean 39); social service home carers: 17-56 years (mean 38); patients: 75-98 years (mean 82), all frail	District nurses: 14 female, 1 male; social service carers: 12 female, 3 male; patients: not fully specified, but presumably mixed
Lora Humphrey Beebe (2001) [9]	United States	True experimental	37 (22 experimental, 15 control after exclusions)	18-68 years (mean: 40.4 ± 10.7)	27% female (n = 10), 73% male (n = 27)
Timothy Kwok (2004) [10]	Hong Kong	RCT	157 randomized (77 intervention, 80 control); 140 completed (67 intervention, 73 control)	Aged ≥60, mean age ~75 years	~71% male (56/77 in intervention, 55/80 in control)
Helen Edwards (2005) [11]	Australia	RCT	33 total (16 intervention, 17 control)	<60 years: 9.1%, 60-70 years: 21.2%, 71-80 years: 33.3%, 80 years: 36.4%	Male: 51.5%; female: 48.5%
Susan KY Chow (2008) [12]	Hong Kong	Secondary analysis of an RCT	46 (subgroup from the original RCT of 332 patients)	Mean 72.9 years (SD 6.03)	50% male, 50% female
Helen Edwards (2009) [13]	Australia	RCT	67 (intervention group: 34, control group: 33)	10.4% under 60, 22.2% aged 60-70, 32.8% aged 71-80, 32.8% aged 81-90	53.7% male, 46.3% female
Victoria Monay (2010) [14]	United States	Descriptive observational study nested within a community-based randomized trial	Enrolled: 100 adults, nurse visits analyzed: 63 visits involving 33 participants	Mean 59 years (SD = 11)	64% female
Daniel Lucky (2011) [15]	United States	Descriptive evaluation study	958 individuals screened; 170 had high BP readings; complete follow-up data on 124 individuals	19-88 years (mean: 53.19 ± 15.27 years)	58 male, 66 female (among high BP group with complete data)
Utens CMA (2012) [16]	Netherlands	RCT	139 patients randomized	Mean age approximately 68 years	Usual hospital care: 55.1% male; early assisted discharge: 68.6% male
Mark F. Harris (2013) [17]	Australia	Quasi-experimental trial	804 clients recruited (from 2361 eligible, 34.1% participation rate)	30-80 years (67.1% aged ≥60)	49.3% female
Ippoliti R (2018) [18]	Italy	Economic and policy evaluation break-even analysis within a program evaluation framework	191,977 elderly individuals in mountain areas of Piedmont Region	65 years and older	Not specified
Yi Xu (2022) [19]	Singapore	Single-group pretest-posttest study	1600	Median 71 years (IQR 64-79), aged ≥60	41% male, 59% female
Rute Rego (2023) [20]	Portugal	Quantitative original article; descriptive and exploratory study based on a health planning methodology	18 users + 5 family members (convenience sample)	Mean 73.61 years (range: 55-88 years)	67% male
Soraia Nobre Figueiredo (2023) [21]	Portugal	Quantitative original article; analytical cross-sectional observational study with community intervention	30 individuals with hypertension and their families	Mean age 56.27 years (SD = 6.38); inclusion: 45-65 years old	47% male
Marzieh Moattari (2023) [22]	Canada	Quality improvement project	Chart review: 157 chart (pre: 93, post: 64); eligible charts: 12 pre, 10 post; QoL self-assessment participants: 17 clients (pre: 11, post: 6)	Mean age: 68.3 years (pre), 76 years (post)	59% female, 41% male
Andreja Ljubič (2023) [23]	Slovenia	Qualitative comparative case study	4 older adults (2 with dementia, 2 without)	82-91 years	1 male, 3 female
Melanie Thomas (2024) [24]	United Kingdom	Observational before-and-after study	561 patients completed the evaluation	Mean age 70 years (range 42-101)	Female: 59%

Definitions of Community Nursing

The concept of community nursing was defined variably across the included studies, reflecting both national healthcare structures and local implementation contexts [5,6,19,20]. Despite these differences, a shared conceptual foundation emerged, centered on delivering preventive, supportive, and patient-centered care outside of hospital settings [5,6,19].

In several studies, particularly from Singapore and Australia, community nursing has been positioned as an extension of primary care, offering a nurse-led model embedded within the local community [17,19]. These services often included health risk assessments, care planning, health coaching, and structured follow-up to reduce avoidable hospitalizations and support aging in place [17,19,20].

Studies from Southern European countries, such as Portugal and Italy, have highlighted community nursing as a specialized public health intervention [18,20,21]. In these contexts, community nursing involved delivering structured education, behavior change support, and family involvement in chronic disease management. Theoretical models, including the Ottawa Charter, the Health Promotion Planning Model, and self-care theories, often guided interventions [20,21].

In the United States, community nursing is often described in more population-based terms, with implementation frequently facilitated through partnerships among public health departments, law enforcement agencies, and community organizations [14,15]. This model prioritized health screenings, risk stratification, and early identification of unmet health needs in underserved populations (Table 3) [14,15].

**Table 3:** Definitions and characteristics of community nursing across regions This table summarizes how community nursing was defined and operationalized in different national contexts across the included studies. While terminology and implementation varied, common elements such as preventive care, community proximity, and patient empowerment were consistently observed. Theoretical models were explicitly referenced in some settings but not uniformly across all studies.

Country/Region	Definition Focus	Key Features	Theoretical Frameworks
Singapore, Australia	Primary care extension; nurse-led local model	Health risk assessments, coaching, care coordination, follow-up to reduce hospitalizations [17,19]	Not explicitly stated; operational model emphasized
Portugal, Italy	Specialized public health intervention	Structured education, behavioral support, family involvement; guided by health promotion theories [18,20,21]	Ottawa Charter, health promotion planning model, self-care theories [20,21]
United States	Population-level public health outreach	Health screenings, risk stratification, inter-sectoral collaboration for underserved populations [14,15]	Not specified; aligned with public health systems

Roles of Community Nurse

The role and qualifications of the community nurse also varied significantly across the studies, influenced by local regulatory frameworks, professional scopes, and healthcare workforce structures.

In some contexts, such as Singapore and parts of Europe, the community nurse was described as an advanced practice nurse (APN), such as a nurse practitioner or clinical nurse specialist, who was trained to conduct independent assessments, lead care coordination, and implement complex interventions. These nurses often led multidisciplinary teams and had prescriptive authority or extended clinical responsibilities.

In contrast, several studies, particularly from Australia and Portugal, referred to community nurses as generalist registered nurses practicing within defined community health networks. These nurses provided basic health education, lifestyle counseling, medication support, and monitoring for patients with chronic illnesses, often in collaboration with physicians and allied health professionals.

In Italy, community nurses were sometimes described as having specialized training (e.g., in health promotion and cardiovascular risk management), functioning in roles akin to public health educators or patient empowerment facilitators. Their work included structured workshops, development of self-care tools, and ongoing behavioral coaching for patients and families.

In the United States, community nurses (often public health nurses) played a dual role: delivering population-level preventive services (e.g., blood pressure screenings) and connecting individuals to clinical care pathways. Their practice was embedded in a broader framework of public safety and health system navigation. Common elements across all definitions of a community nurse included autonomous or semi-autonomous practice within community settings, provision of health education, prevention, and early intervention, an active role in care coordination and multidisciplinary collaboration, and cultural competence and the ability to engage with socially diverse populations (Table 4).

**Table 4:** Definitions, roles, and attributes of community nurses across regions This table illustrates the variation in community nurse roles and qualifications across geographic settings. While the scope of practice ranged from generalist to advanced practice levels, common features included autonomous care delivery, health education, care coordination, and cultural competence within community-based settings.

Country/Region	Professional Level	Roles and Responsibilities	Common Attributes
Singapore, parts of Europe	Advanced practice nurse (e.g., nurse practitioner and clinical nurse specialist)	Independent assessments, care coordination, complex interventions, leadership of multidisciplinary teams, prescriptive authority [17,19]	Autonomous practice; team leadership [17,19]
Australia, Portugal	Generalist registered nurse	Health education, lifestyle counseling, medication support, chronic illness monitoring, collaborative work with general practitioners and allied professionals [17,20,21]	Basic education and support within care networks [20,21]
Italy	Specialized community nurse/public health educator	Health promotion education, behavioral coaching, development of self-care tools, family engagement [18,20]	Empowerment-focused, structured workshops [18,20]
United States	Public health nurse	Population-level screening, public health outreach, system navigation, linkage to care pathways [14,15]	Dual public health and clinical linkage role [14,15]

Description of Interventions

Community nursing interventions included in the reviewed studies were diverse in structure, duration, and delivery settings. Despite this variation, six core components consistently emerged, reflecting a shared focus on preventive care, chronic disease management, and patient empowerment.

Health assessments: Most interventions began with comprehensive assessments that included vital signs, frailty, nutritional status, and social risk factors. These were used to tailor care plans and were sometimes repeated to monitor progress.

Health coaching and lifestyle education: Nurses provided individualized or group-based coaching on diet, physical activity, substance use, and stress management. Behavior change theories (e.g., social cognitive theory) informed many interventions.

Self-management and medication support: Patients were trained to self-monitor their conditions (e.g., hypertension), adhere to treatment plans, and overcome medication-related challenges, often using tools such as diaries or pill organizers.

Caregiver and family education: Several programs involved family members in structured education to support patient behavior and reduce caregiver burden, particularly emphasized in Southern Europe.

Community nurse training: Some studies focused on building nurse competencies through structured programs, such as an 18-month course in Italy on chronic disease prevention and health promotion.

Duration and delivery setting: Intervention lengths ranged from three-day outreach fairs to 18-month programs. Delivery formats included home visits, community health centers, and mobile or public space-based services.

These components are summarized in Table 5.

**Table 5:** Key components of community nursing interventions across studies This table summarizes the core components of community nursing interventions observed across the included studies, highlighting their diversity in structure, duration, and delivery settings. Despite variation, most interventions emphasized patient-centered care, chronic disease support, and health education.

Intervention Component	Description
Health Assessments	Initial and periodic assessments: blood pressure, frailty, nutrition, risk screening [17,19,20]
Health Coaching & Lifestyle Education	Patient-centered counseling on diet, exercise, substance use, stress; often using behavior change theories [17,20,21]
Self-Management & Medication Support	Support for chronic disease management and medication adherence (e.g., BP monitoring, education, coordination) [19-21]
Caregiver & Family Education	Family inclusion in structured sessions to reinforce behavior and reduce caregiver burden [18,20,21]
Community Nurse Training Programs	Programs to build nurse competencies in health promotion and chronic care [18,20]
Intervention Duration & Setting	3 days to 18 months; delivered via home visits, community centers, or outreach events [14,15,18-20]

Outcomes Measured and Effectiveness of Community Nursing Interventions

The included studies evaluated a broad range of outcomes across four domains: (1) healthcare utilization, (2) patient-reported outcomes (PROs), (3) clinical indicators, and (4) quality of life and satisfaction. While designs and measures varied, most studies used pre/post comparisons, with limited use of control groups.

Healthcare service utilization: Several interventions reduced ED visits and hospital admissions. For example, the Singapore program (n = 1,600) reported a 23% decrease in ED use (IRR: 0.77, 95% CI: 0.71-0.83, p < 0.001) [19]. In addition to this, studies from Italy [18], Australia [17], and Hong Kong [10,12] also showed reductions in unplanned healthcare utilization or improvements in chronic disease self-management. However, effect sizes and confidence intervals were not consistently reported across studies, limiting formal synthesis.

Patient-reported outcomes: Several studies reported improvements in self-efficacy, health knowledge, and adherence to healthy behaviors such as diet, physical activity, and medication use. In Portugal, patients and caregivers participating in community nursing interventions showed greater confidence in disease management and self-monitoring [20,21]. Similar improvements in health literacy and behavior were reported in Australia [13,17], Hong Kong [12], and Canada [22]. In the United States, brief interventions increased awareness and motivation to seek care [14,15]. A Greek training program also improved nurses’ perceived competencies (from 5.8 to 8.2 on a 10-point scale, p < 0.001) [21].

Clinical indicators: Most studies focused on hypertension-related outcomes, with several reporting improved blood pressure monitoring or control. For example, a Portuguese study increased regular home blood pressure monitoring from 0% to 70% following a structured intervention [21]. In Hong Kong, Chow et al. [12] observed a significant reduction in systolic blood pressure (mean decrease: -7.8 mmHg, 95% CI: -12.1 to -3.5, p < 0.01) among post-discharge patients receiving community nursing care. Other clinical indicators, such as body mass index (BMI), glycemic control (HbA1c), and wound healing, were reported less frequently and with limited standardization. For instance, Moattari et al. [22] in Canada described qualitative improvements in wound care outcomes. At the same time, Edwards et al. [11,13] in Australia noted better healing rates of chronic leg ulcers in intervention groups.

Quality of life and satisfaction: Several studies reported improvements in psychological well-being, patient empowerment, and satisfaction with care. In Australia, Edwards et al. [13] observed improved quality of life scores (e.g., SF-36) and leg ulcer healing outcomes following community nursing interventions. In Canada, Moattari et al. [22] noted that clients felt more confident and supported in managing complex care needs at home. Interventions that involved family members or caregivers, particularly in Portugal and Italy, were associated with greater caregiver knowledge, reduced emotional burden, and improved care satisfaction [20,21]. Similarly, programs in the United States [14] and Singapore [19] highlighted positive perceptions of nurse-patient relationships and trust in community-based care.

These findings are summarized in Table 6.

**Table 6:** Summary of outcomes and reported effectiveness This table summarizes the primary outcome domains assessed across included studies, highlighting representative findings and study settings. While most studies reported positive trends in patient- and system-level outcomes, the evidence base is limited by methodological variability, small sample sizes, and reliance on self-reported measures. PROs, patient-reported outcomes

Outcome Domain	Representative Findings	Example Study/Setting
Healthcare Utilization	23% reduction in ED visits (IRR 0.77, p < 0.001); fewer unplanned admissions [19]	Singapore, large cohort (n = 1600) [19]
PROs	Improved self-efficacy, health knowledge, lifestyle adherence (e.g., diet, activity) [20,21]	Italy (nurse training), Portugal (structured education) [18,20,21]
Clinical Indicators	Increase in BP self-monitoring (0%→70%); limited data on other biomarkers [21]	Portugal, hypertension-focused intervention [21]
Quality of Life & Satisfaction	Improved psychological well-being, patient empowerment, caregiver support [18,20,21]	Southern Europe, caregiver-inclusive models [18,20,21]

Risk of Bias Assessment Based on ROBINS-I

The risk of bias for the 17 included non-randomized studies was assessed using the ROBINS-I tool. This framework evaluates seven domains of bias in observational and quasi-experimental intervention studies, allowing for a structured comparison of methodological quality.

Bias due to confounding: High risk of bias was observed in most studies in this domain. Most studies did not account for key confounders such as baseline health status, socioeconomic conditions, or comorbidity burden. None employed techniques such as propensity score matching, regression adjustment, or stratified analysis to reduce confounding. As a result, the observed intervention effects may have been influenced by unmeasured differences between participants.

Bias in selection of participants into the study: The risk of selection bias was assessed as moderate to high. Several studies used convenience samples from clinics or community centers without clearly defining eligibility criteria. Random sampling or systematic recruitment methods were rarely applied. Only two studies used comparison groups, and even in those, allocation was not randomized, increasing the likelihood of baseline imbalances.

Bias in classification of interventions: This domain showed low-to-moderate risk. Most studies provided a clear description of the community nursing intervention, including its components and duration. However, some lacked detail on how interventions were implemented consistently across participants. There was little evidence of misclassification of intervention status, although fidelity assessment was rarely reported.

Bias due to deviations from intended interventions: This domain was difficult to evaluate comprehensively due to the limited reporting on intervention fidelity and adherence. No studies reported protocol deviations or participant noncompliance. Consequently, this domain was generally rated as moderate risk, based on uncertainty.

Bias due to missing data: This domain was rated as moderate risk in most studies. While follow-up rates were not always reported, several studies failed to account for attrition or missing outcome data. Intention-to-treat analysis was not applied. In smaller studies, even a modest loss to follow-up can significantly impact the results.

Bias in measurement of outcomes: This domain was frequently rated as moderate to high risk. Several outcomes, including self-efficacy, satisfaction, and health behaviors, were assessed through self-reported questionnaires, which the authors often developed without validation. Objective outcomes (e.g., blood pressure) were infrequently reported and seldom verified independently.

Bias in selection of the reported result: Most studies appeared to report results in full, and there was little evidence of selective reporting. This domain was rated as low-to-moderate risk across studies, although the absence of published protocols made definitive assessment challenging (Table 7).

**Table 7:** ROBINS-I risk of bias assessment for included non-randomized studies ROBINS-I, Risk Of Bias In Non-randomized Studies - of Interventions

Study	Confounding	Selection of Participants	Classification of Interventions	Deviations From Intended Interventions	Missing Data	Measurement of Outcomes	Selection of Reported Result
Flanagan et al. (2001) [8]	High	Moderate	Low/Moderate	Moderate	Moderate	Moderate/High	Low/Moderate
Beebe (2001) [9]	High	Moderate	Low/Moderate	Moderate	Moderate	Moderate/High	Low/Moderate
Kwok et al. (2004) [10]	High	Moderate	Low/Moderate	Moderate	Moderate	Moderate/High	Low/Moderate
Edwards et al. (2005) [11]	High	Moderate	Low/Moderate	Moderate	Moderate	Moderate/High	Low/Moderate
Chow et al. (2008) [12]	High	Moderate	Low/Moderate	Moderate	Moderate	Moderate/High	Low/Moderate
Edwards et al. (2009) [13]	High	Moderate	Low/Moderate	Moderate	Moderate	Moderate/High	Low/Moderate
Monay et al. (2010) [14]	High	Moderate	Low/Moderate	Moderate	Moderate	Moderate/High	Low/Moderate
Lucky et al. (2011) [15]	High	Moderate	Low/Moderate	Moderate	Moderate	Moderate/High	Low/Moderate
Utens et al. (2012) [16]	High	Moderate	Low/Moderate	Moderate	Moderate	Moderate/High	Low/Moderate
Harris et al. (2013) [17]	High	Moderate	Low/Moderate	Moderate	Moderate	Moderate/High	Low/Moderate
Ippoliti et al. (2018) [18]	High	Moderate	Low/Moderate	Moderate	Moderate	Moderate/High	Low/Moderate
Xu et al. (2022) [19]	High	Moderate	Low/Moderate	Moderate	Moderate	Moderate/High	Low/Moderate
Rego et al. (2023) [20]	High	Moderate	Low/Moderate	Moderate	Moderate	Moderate/High	Low/Moderate
Nobre Figueiredo et al. (2023) [21]	High	Moderate	Low/Moderate	Moderate	Moderate	Moderate/High	Low/Moderate
Moattari et al. (2023) [22]	High	Moderate	Low/Moderate	Moderate	Moderate	Moderate/High	Low/Moderate
Ljubič et al. (2023) [23]	High	Moderate	Low/Moderate	Moderate	Moderate	Moderate/High	Low/Moderate
Thomas et al. (2024) [24]	High	Moderate	Low/Moderate	Moderate	Moderate	Moderate/High	Low/Moderate

Discussion

Summary of the Study

This systematic review synthesized the characteristics, components, and effectiveness of community nursing interventions designed to promote health, manage chronic diseases, and enhance quality of life among community-dwelling populations. Drawing upon studies conducted across diverse international contexts, including Singapore, Portugal, Italy, Australia, and the United States, we identified six core intervention components: health assessments, health coaching, self-management support, caregiver education, nurse training, and varied delivery formats. Outcomes were categorized into four domains: healthcare service utilization, PROs, clinical indicators, and quality of life.

Across most studies, community nursing interventions demonstrated promising results, including reduced ED visits, improved self-efficacy, better chronic disease self-monitoring, and enhanced patient satisfaction. Despite heterogeneity in intervention designs, durations, and outcome metrics, common trends suggested that community nurses play a pivotal role in bridging gaps between healthcare systems and communities, especially for older adults and those with chronic conditions.

Comparison With Other Studies

The findings of this review are consistent with previous literature underscoring the importance of community-based, nurse-led interventions in chronic disease management. For example, a Cochrane review on home-based nursing for heart failure reported reductions in hospital readmission and improved patient satisfaction, similar to the trends observed in our review [25]. Likewise, studies in integrated care models, such as those in Canada and the UK, have highlighted how community nurses contribute to better care continuity and patient empowerment through sustained, personalized interactions [22,26].

Notably, our review also revealed regional nuances in how community nursing is conceptualized and operationalized. For instance, while community nurses in Singapore often function as advanced practice providers with extended clinical roles, those in Southern Europe are more aligned with public health education and behavioral coaching [19]. These differences reflect variations in healthcare infrastructure and scope of nursing practice, yet the effectiveness of interventions across regions emphasizes the adaptability and value of community nursing.

However, it is also important to note the lack of evidence from many regions, including large parts of Africa, South America, and Southeast Asia beyond Singapore. This underrepresentation may be due to limitations in research funding, publication capacity, or differences in how community nursing is defined, implemented, or documented in those healthcare systems. Addressing these gaps in future studies will be essential for a truly global understanding of community nursing models.

Strengths of the Study

A significant strength of this review lies in its comprehensive synthesis of international evidence, encompassing a broad spectrum of community nursing models and intervention types. By organizing interventions into thematic components and linking them to outcome domains, we provide a structured understanding that can inform practice and policy. The inclusion of nurse training programs as part of the intervention landscape also highlights the importance of capacity building and professional development in sustaining high-quality community care [27,28].

Another strength is the focus on both patient- and system-level outcomes. While many reviews focus solely on clinical endpoints, our analysis encompasses health service utilization, behavior change, caregiver support, and satisfaction, key indicators of value-based care and population health improvement [29].

Limitations

Several limitations should be acknowledged. First, most included studies employed pre/post designs without control groups, raising concerns about internal validity and the potential influence of confounding variables. Second, many outcomes were self-reported and lacked standardized measurement tools, which limited comparability across studies and reduced the strength of the evidence.

Furthermore, the heterogeneity in study settings, intervention durations, and professional roles of nurses made meta-analysis infeasible. Some studies did not clearly define the qualifications or scopes of practice of the community nurses involved, which may affect the generalizability of findings to other health systems. Finally, publication bias may exist, as most included studies reported positive results, and studies published in non-English languages or gray literature may have been missed.

Further Directions

This review highlights the potential of community nursing not only as a professional domain but also as a broader framework for community-based health support. Clarifying the roles and functions of community nursing can empower not only trained nurses but also community health workers and laypersons to contribute meaningfully, especially in resource-limited or rural settings [27,28]. In such environments, where access to healthcare professionals is often restricted, the collaboration between community nurses and local workers may be essential to addressing issues such as social isolation, loneliness, and chronic disease management, particularly among indigenous and aging populations [30]. These challenges are not limited to low-resource settings; aging societies in high-income countries also face similar dynamics, with younger populations migrating to urban centers and older adults left behind with reduced support networks. Future research should explore models that integrate community members, such as volunteers, peer educators, and caregivers, into the design and delivery of community nursing interventions. Engaging a broader base of stakeholders may enhance the reach, sustainability, and cultural relevance of care, ultimately contributing to stronger, healthier communities. Promoting such inclusive approaches will be essential to building resilient health systems and fostering community well-being in the face of demographic and social change.

## Conclusions

Community nursing interventions offer a versatile and practical approach to delivering patient-centered care in non-hospital settings. To strengthen the global evidence base, there is a need for internationally standardized reporting of intervention components, outcome measures, and clearly defined nursing roles. Such standardization would improve comparability across studies, facilitate best practice sharing, and support the scaling of effective community-based models. Despite variations in structure and delivery, common elements, such as health education, chronic disease support, and community engagement, were consistently linked to improved outcomes. The integration of community nurses into primary care and public health systems can help reduce healthcare utilization, improve self-management, and enhance patient satisfaction. Future research should focus on high-quality randomized controlled trials using standardized outcome measures and clearly defined nursing roles. Such evidence is essential for guiding policy decisions and expanding the implementation of community nursing models that are both scalable and culturally adaptable.

## References

[1] Sabir M, Wethington E, Breckman R, Meador R, Reid MC, Pillemer K (2009). A community-based participatory critique of social isolation intervention research for community-dwelling older adults. J Appl Gerontol.

[2] Vetrano DL, Palmer K, Marengoni A (2019). Frailty and multimorbidity: a systematic review and meta-analysis. J Gerontol A Biol Sci Med Sci.

[3] Ohta R, Maejma S, Sano C (2022). Nurses' contributions in rural family medicine education: a mixed-method approach. Int J Environ Res Public Health.

[4] Leung AY, Su JJ, Lee ES, Fung JT, Molassiotis A (2022). Intrinsic capacity of older people in the community using WHO Integrated Care for Older People (ICOPE) framework: a cross-sectional study. BMC Geriatr.

[5] Zeydani A, Atashzadeh-Shoorideh F, Hosseini M, Zohari-Anboohi S (2023). Community-based nursing: a concept analysis with Walker and Avant's approach. BMC Med Educ.

[6] McBride M, Kilgore C, Oozageer Gunowa N (2024). The role of community and district nurses. Clin Integr Care.

[7] Page MJ, McKenzie JE, Bossuyt PM (2021). The PRISMA 2020 statement: an updated guideline for reporting systematic reviews. BMJ.

[8] Flanagan M, Rotchell L, Fletcher J, Schofield J (2001). Community nurses', home carers' and patients' perceptions of factors affecting venous leg ulcer recurrence and management of services. J Nurs Manag.

[9] Beebe LH (2001). Community nursing support for clients with schizophrenia. Arch Psychiatr Nurs.

[10] Kwok T, Lum CM, Chan HS, Ma HM, Lee D, Woo J (2004). A randomized, controlled trial of an intensive community nurse-supported discharge program in preventing hospital readmissions of older patients with chronic lung disease. J Am Geriatr Soc.

[11] Edwards H, Courtney M, Finlayson K, Lewis C, Lindsay E, Dumble J (2005). Improved healing rates for chronic venous leg ulcers: pilot study results from a randomized controlled trial of a community nursing intervention. Int J Nurs Pract.

[12] Chow SK, Wong FK, Chan TM, Chung LY, Chang KK, Lee RP (2008). Community nursing services for postdischarge chronically ill patients. J Clin Nurs.

[13] Edwards H, Courtney M, Finlayson K, Shuter P, Lindsay E (2009). A randomised controlled trial of a community nursing intervention: improved quality of life and healing for clients with chronic leg ulcers. J Clin Nurs.

[14] Monay V, Mangione CM, Sorrell-Thompson A, Baig AA (2010). Services delivered by faith-community nurses to individuals with elevated blood pressure. Public Health Nurs.

[15] Lucky D, Turner B, Hall M, Lefaver S, de Werk A (2011). Blood pressure screenings through community nursing health fairs: motivating individuals to seek health care follow-up. J Community Health Nurs.

[16] Utens CM, Goossens LM, Smeenk FW (2012). Early assisted discharge with generic community nursing for chronic obstructive pulmonary disease exacerbations: results of a randomised controlled trial. BMJ Open.

[17] Harris MF, Chan BC, Laws RA (2013). The impact of a brief lifestyle intervention delivered by generalist community nurses (CN SNAP trial). BMC Public Health.

[18] Ippoliti R, Allievi I, Falavigna G (2018). The sustainability of a community nurses programme aimed at supporting active ageing in mountain areas. Int J Health Plann Manage.

[19] Xu Y, Koh XH, Chua YT (2022). The impact of community nursing program on healthcare utilization: a program evaluation. Geriatr Nurs.

[20] Rego R, Sousa E, Pinto F (2023). Training hypocoagulated users and their families in disease management: a community nursing intervention. Pensar Enferm.

[21] Nobre Figueiredo S, Brites MJ, Sousa JE (2023). Empowerment of hypertensive individuals and families in disease management: a community nursing intervention. Pensar Enferm.

[22] Moattari M, King EC, Ruco A (2023). Whole versus hole: enabling community nurses to implement holistic wound care. J Wound Care.

[23] Ljubič A, Hozjan D, Filej B, Kolnik T (2023). Montessori activities for older adults in community nursing: comparative case study. Nurs 21st Century.

[24] Thomas M, Morgan K, Humphreys I (2024). Lymphoedema specialists embedded into community nurse and wound services: impacts and outcomes. Br J Nurs.

[25] Kalhor F, Shahzeydi A, Taghadosi M (2024). The impact of home care on individuals with chronic heart failure: a comprehensive review. ARYA Atheroscler.

[26] Siette J, Dodds L, Surian D, Prgomet M, Dunn A, Westbrook J (2022). Social interactions and quality of life of residents in aged care facilities: a multi-methods study. PLoS One.

[27] Ohta R, Maiguma K, Yata A, Sano C (2022). A solution for loneliness in rural populations: the effects of Osekkai conferences during the COVID-19 pandemic. Int J Environ Res Public Health.

[28] Herai R, Ohta R, Sano C (2023). Reviving health Osekkai in rural Japan: collaborative strategies of family physicians and medical students against social isolation. Cureus.

[29] Ohta R, Maiguma K, Yata A, Sano C (2022). Rebuilding social capital through Osekkai conferences in rural communities: a social network analysis. Int J Environ Res Public Health.

[30] Naito Y, Ohta R, Sano C (2021). Solving social problems in aging rural Japanese communities: the development and sustainability of the Osekkai conference as a social prescribing during the COVID-19 pandemic. Int J Environ Res Public Health.

